# Research note reliability and validity of Japanese version of the trauma-informed care provider survey (TIC provider survey)

**DOI:** 10.1186/s13104-023-06337-8

**Published:** 2023-05-02

**Authors:** Mayumi Kataoka, Risa Kotake, Hiroki Asaoka, Yuki Miyamoto, Daisuke Nishi

**Affiliations:** 1 Department of Mental Health, Graduate School of Medicine, The University of Tokyo, 7-3-1 Hongo, Bunkyo-ku, Tokyo, 113-0033 Japan; 2 Department of Public Mental Health Research, National Institute of Mental Health, National Center of Neurology and Psychiatry, 4-1-1 Ogawahigashicho, Kodaira, Tokyo, 187-8553 Japan; 3 Department of Psychiatric Nursing, Graduate School of Medicine, The University of Tokyo, 7- 3-1 Hongo, Bunkyo-ku, Tokyo, 113-0033 Japan

**Keywords:** Scale, Trauma, Trauma-informed care, Reliability, Validity

## Abstract

**Objective:**

Robust instruments to evaluate the ability of trauma-informed care among healthcare workers need to be developed, as this would help the implementation of trauma-informed care to prevent re-traumatization of patients. This study aims to assess the reliability and validity of the Japanese version of the Trauma-Informed Care (TIC) Provider Survey. A total of 794 healthcare workers were surveyed using a self-administered questionnaire, including the TIC Provider Survey, and six measures that were considered to be correlated with it. We calculated the Cronbach’s alpha coefficient to investigate the internal consistency of each category of the TIC Provider Survey (knowledge, opinions, self-rated competence, practices, and barriers). Spearman’s rank correlation coefficients were used to investigate the correlation between each category of the TIC Provider Survey, and other measures of construct validity.

**Results:**

Cronbach’s alpha coefficients of each category of the TIC Provider Survey were 0.40 (Knowledge), 0.63 (Opinions), 0.92 (Self-rated competence), 0.93 (Practices), and 0.87 (Barriers). The Spearman’s rank correlation coefficients were small. We confirmed the reliability of the acceptable levels and examined the validity of modest or unacceptable levels of the Japanese version of the TIC provider survey among Japanese workers in a healthcare setting.

**Supplementary Information:**

The online version contains supplementary material available at 10.1186/s13104-023-06337-8.

## Introduction

The trauma-informed care (TIC) refers to the approach that acknowledges the presence of trauma and its effects on patients and those involved in caregiving to prevent re-traumatization among these individuals in a sensitive and considerate manner [1]. Many patients in healthcare settings experience trauma. For example, 94% of inpatients in psychiatric hospitals have experienced at least one traumatic event in their lives [2]. Patients with traumatic experiences are likely to suffer from re-traumatization even with usual medical care [3–5]. Patients’ re-traumatization has been considered a problem because it increases the risk of developing psychosocial issues and poor health outcomes for both, patients and healthcare workers [6–8]. Therefore, it is important to prevent re-traumatization in healthcare settings. One approach to prevent re-traumatization among healthcare settings is TIC [1, 4, 8]. Previous research showed a decrease of 82.3% in the rate of re-traumatizing medical practices, such as seclusion and restraint with TIC [9]. It is considered that TIC reduces re-traumatization of patients and lessens stress levels of healthcare workers, increasing their job satisfaction [10, 11]. TIC is being considered useful for the safety of both patients and workers in healthcare settings in Japan [12].

However, certain barriers exist in implementing TIC in healthcare settings, one being the lack of robust instruments to evaluate its ability [13]. The original self-report scale, the Trauma-informed Care Provider Survey (TIC Provider Survey), was developed to assess Knowledge, Opinions favorable to trauma-informed care, Self-rated competence, recent Practice, and perceived Barriers to TIC; its reliability was confirmed [14, 15].

We developed a Japanese version of the TIC provider survey with the original developer’s support. This study aimed to confirm its reliability and validity in a sample of workers in a health care setting.

## Methods

### Participants

We recruited 1000 employees working at hospitals, clinics, and health care centers, through a pooled panels of an internet research agency in Japan (Rakuten Insight, Inc.), which had approximately 2.2 million panelists in 2019. In the pooled panels, only healthcare workers in medical institutions or health organization were asked to cooperate in the survey. Those who agreed were then invited to participate. All the participants provided web-based informed consent at registration and accessed the questionnaires on the website, and responded to the questions in November 2020. The inclusion criteria were, (1) age over 18 years, and (2) health care workers in Japan. After excluding the administrative staff (n = 206), 794 participants (response rate = 79.4%) were considered for the analysis. The study was in accordance with the Declaration of Helsinki. All methods were performed in accordance with the relevant guidelines and regulations.

## Measurements

### Trauma-informed care provider survey (TIC provider survey)

The original version of the TIC Provider Survey (version for providers caring for adult patients) was a 38-item self-administered questionnaire measuring the key elements and practices of TIC, consisting of the following five categories: 1) knowledge about trauma-informed care, with 11 items (Knowledge); 2) opinions about trauma-informed care, with 6 items (Opinions); 3) self-rated competence, with10 items (Self-rated competence); 4) recent practice, with 7 items (Practices); 5) perceived barriers to implementation of trauma-informed care, with 4 items (Barriers). Knowledge and Opinions are rated on a 4-point Likert scale; Self-rated competence and Barriers on a 3-point Likert scale. Practice is a binary variable with yes [1], and no (0). The internal consistency (Cronbach’s alpha) of the original version ranged from fair to excellent (e.g., Knowledge (0.49), Opinions (0.67), Self-rated competence (0.90), Practices (0.83), and Barriers (.70)) [16]. The total scores for Knowledge, Opinions, Self-rated competence, and Practices were summed up, with each category range as follows (Knowledge, 11–44 ; Opinions, 6–24; Self-rated competence, 0–20; Practices, 0–7). Higher scores indicate greater knowledge, more favorable opinions, greater self-rated competence, and more frequent practice of TIC. With the original authors’ permission, we divided one item of Barriers, i.e., “time and practice constraints,” into two: ”time constraints,” and ” practice constraints”. The total number of items in the Japanese version was 39.

With the authors’ permission, we translated the original version of the TIC Provider Survey into Japanese. We followed the standard back-translation procedure. Two authors (D.N. and Y.M.) translated the scale into Japanese as the draft of the Japanese version. Plain Japanese was used in the translation. The draft was revised after receiving feedback from five mental health professionals in Japan. This draft of the Japanese version was translated back into English by an independent translator. The back-translated version was examined by Dr. Nancy Kassam-Adams and Dr. Therese S. Richmond, who had developed the original version. Then three authors (D.N., Y.M., and R.K.) amended the Japanese translation accordingly. The developers of the original TIC Provider Survey reviewed and approved the final back-translated version of the revised Japanese version.

### Other measures

The construct validity of the TIC Provider Survey was tested against other scales assumed to be correlated with it.

### Japanese version of the attitude-related trauma-informed care scale (ARTIC-10)

The ARTIC-10 is a validated self-administered questionnaire that assesses attitudes towards TIC implementation [13, 17]. We used the Japanese version of the ARTIC-10, created using back translation [12]. We hypothesized that the TIC Provider Survey would positively correlate with ARTIC-10, because favorable attitudes towards TIC are similar to Knowledge, Opinions, Self-rated competence, and Practice of the TIC Provider Survey.

### The Japanese version of the moral sensitivity questionnaire 2018 (J-MSQ 2018)

The J-MSQ 2018 is a validated self-administered questionnaire measuring moral sensitivity [18, 19]. We hypothesized that the TIC Provider Survey would positively correlate with J-MSQ 2018 because moral sensitivity is defined as a genuine concern for another’s welfare, [20] and is similar to the concept of TIC, integrating knowledge about trauma into practice, to resist re-traumatization of trauma survivors [1].

### Patient health questionnaire-9 (PHQ-9)

The Patient Health Questionnaire-9 (PHQ-9) is a validated self-administered questionnaire developed to assess the frequency of symptoms of depression that occurred in the prior two weeks [21].

### Generalized anxiety disorder-7 (GAD-7)

The GAD-7 is a validated self-administered questionnaire developed to assess the frequency of anxiety symptoms that occurred in the previous two weeks [22].

We hypothesized that the TIC Provider Survey would negatively correlate with the PHQ-9 and GAD-7 because a previous study showed that higher scores on the ARTIC-10 (similar to the TIC Provider Survey) negatively correlated with burnout and secondary traumatic stress (STS) [17].

### Stress underestimation beliefs (SUB)

The SUB is a validated self-administered questionnaire, developed to measure Japanese stress underestimation beliefs [23]. We hypothesized that the TIC Provider Survey would negatively correlate with SUB because a previous study showed that respondents with stress-related symptoms were likely to have more stress underestimation beliefs [24].

### Negative acts questionnaire-revised (NAQ-R)

The NAQ-R is a validated self-administered questionnaire, developed to measure workplace bullying, and the frequency with which participants experienced it during the previous six months [25]. We hypothesized that the TIC Provider Survey would negatively correlate with NAQ-R because previous studies have shown that individuals with higher clinical abilities are less likely to experience workplace bullying [26].

### Demographic variables

The assessed demographic variables included gender, age, marital status, educational level, job category, and years of work experience.

### Statistical analysis

To examine internal consistency, Cronbach’s alpha was calculated for each category of the Japanese version of the TIC Provider Survey. To assess construct validity, Spearman’s rank correlation coefficients were calculated between the total score of each category of the TIC Provider Survey and the following six variables: ARTIC-10, PHQ-9, GAD-7, J-MSQ2018, SUB, and NAQ-R. We selected Spearman’s rank correlation coefficients because the normality test indicated that the scores of the scales were not distributed normally. All analyses were performed using Stata 16.1 (StataCorp LLC, USA).

## Results

### Characteristics of the participants

Table 1 shows the participants’ characteristics, median values, and interquartile range (IQR). The median age and years of work experience were 42 (IQR, 34–52) and 16 (IQR, 9–25), respectively. Regarding demographic characteristics, 45.6% were male, 54.4% were female, 36.0% were physicians, and 64.0% were nurses (including practical nurses). Table 2 shows the scale measurement results of the participants.

**Table 1:** Demographics and characteristics of the participants (n = 794)

Variables		n	%	mean	SD	IQR
Gender						
Female		432	54.4			
Male		362	45.6			
Age				43.1	11.2	42 (34–52)
Marital status						
Married, Common-law marriage		516	65.0			
Never married, Widowed, Divorced		278	35.0			
Educational level						
High school graduate		56	7.1			
Two-year college graduate		247	31.1			
Bachelor’s degree		204	25.7			
Master’s or doctoral degree		200	25.1			
Other		87	11.0			
Job category						
Physician		286	36.0			
Nurse		508	64.0			
Years of work experience				17.7	10.4	16 (9–25)

**Table 2:** Scale measurement results of the participants (n = 794)

Variables		n	%	mean	SD	IQR	Not a barrier	%	Somewhat of a barrier	%	Significant barrier	%
TIC provider survey												
Knowledge				28.6	2.9	29 (27–30)						
Opinions				16.4	2.4	17 (15–18)						
Self-rated competence				9.3	4.3	10 (7–10)						
Practices				3.2	2.9	3 (0–7)						
Barriers	1. Time constraints						129	16.2	454	57.2	211	26.6
	2. Scope of practice constraints						153	19.3	462	58.2	179	22.5
	3. Lack of training						182	23.0	464	58.4	148	18.6
	4. Confusing or unclear information on trauma informed care						131	16.5	489	61.6	174	21.9
	5. Worry about further upsetting or traumatizing patients						155	19.5	498	62.7	141	17.8
ARTIC-10				4.3	0.6	4.2 (4-4.7)						
J-MSQ2018				37.5	10.5	39 (32–44)						
PHQ-9				4.1	5.4	2 (0–6)						
GAD-7				3.0	4.5	1 (0–4)						
SUB				27.6	7.7	27 (22–32)						
NAQ-R				27.9	13.1	22 (21–27)						

Note: TIC provider survey: Japanese version of the Trauma-Informed Care Provider Survey; ARTIC-10: The short version of Attitude-related Trauma-Informed Care Scale; J-MSQ_2018: The Japanese version of the Moral Sensitivity Questionnaire 2018; PHQ-9: Patient Health Questionnaire-9; GAD-7: Generalized Anxiety Disorder-7; SUB: Stress Underestimation Beliefs; NAQ-R: Negative Acts Questionnaire-Revised

ARTIC-10 consists of 10 items with a 7-point bipolar Likert scale, asking about attitudes towards TIC on their job, during the previous two months. The mean scores ranged from 1 to 7. Higher scores indicate a more favorable attitude towards TIC

The J-MSQ_2018 consists of 10 items with a 6-point scale, ranging from 1 (total disagreement) to 6 (total agreement). The total scores ranged from 10 to 60. Higher scores indicate higher moral sensitivity

The PHQ-9 consists of 9 items, rated on a 4-point scale, from 0 (not at all) to 3 (nearly every day). The total scores ranged from 0 to 27. Higher scores indicate more severe depressive symptoms

The GAD-7 consists of 7 items, rated on a 4-point scale, from 0 (not at all) to 3 (nearly every day). The total scores ranged from 0 to 21. Higher scores indicate more severe symptoms of anxiety

The SUB consists of 12 items, with a 4-point scale, ranging from 1 (not applicable) to 4 (applicable). The total scores ranged from 12 to 48. Higher scores indicate greater stress underestimation beliefs

The NAQ-R consists of 23 items, with a 5-point scale, ranging from 1 (never) to 5 (daily). The total scores ranged from 23 to 115. Higher scores indicate more workplace bullying. In this study, we could not use two items that asked about violence or abuse in the workplace and perception of workplace bullying because these questions were assumed to be too sensitive for participants and the research company refused to include them. Therefore, we used 21 items of the NAQ-R and summed the total score, which could have ranged from 21 to 105 in this study

### Reliability of TIC provider survey

As shown in Table 3, the Cronbach’s alpha coefficients for each category of the TIC Provider Survey were 0.40–0.93. The reliability ranges were fair or excellent, except for knowledge.

**Table 3:** Reliability of the TIC provider survey and other scales (n = 794)

Scales	Cronbach’s α
TIC provider survey	
Knowledge	0.40
Opinions	0.63
Self-rated competence	0.92
Practices	0.93
Barriers	0.87
ARTIC-10	0.56
J-MSQ2018	0.94
PHQ-9	0.92
GAD-7	0.94
SUB	0.91
NAQ-R	0.98

Note: TIC provider survey: Japanese version of the Trauma-Informed Care Provider Survey; ARTIC-10: The short version of Attitude-related Trauma-Informed Care Scale; J-MSQ_2018: The Japanese version of the Moral Sensitivity Questionnaire 2018; PHQ-9: Patient Health Questionnaire-9; GAD-7: Generalized Anxiety Disorder-7; SUB: Stress Underestimation Beliefs; NAQ-R: Negative Acts Questionnaire-Revised

### Construct validity of TIC provider survey

The construct validity of the TIC provider survey is presented in Table 4. Unexpectedly, the correlations between the TIC Provider Survey and other measures were not significant, or significant but weak, or the opposite of our hypotheses.

**Table 4:** Spearman’s rank correlation coefficient between the TIC provider survey and other scales (n = 794)

Scales	ARTIC-10		J-MSQ2018		PHQ-9		GAD-7		SUB		NAQ-R	
	r	p-value	r	p-value	r	p-value	r	p-value	r	p-value	r	p-value
Knowledge	0.26	< 0.01	0.34	< 0.01	0.08	0.02	0.05	0.18	-0.16	< 0.01	0.02	0.64
Opinions	0.26	< 0.01	0.32	< 0.01	0.01	0.75	<-0.01	0.99	-0.15	< 0.01	-0.06	0.12
Self-rated competence	0.03	0.48	0.22	< 0.01	0.05	0.16	0.07	0.06	0.12	< 0.01	0.09	< 0.01
Practices	0.03	0.37	0.17	< 0.01	0.09	0.01	0.09	< 0.01	0.01	0.76	0.15	< 0.01

Note: TIC provider survey: The Japanese version of the Trauma-Informed Care Provider Survey; ARTIC-10: The short version of Attitude-related Trauma-Informed Care Scale; J-MSQ_2018: The Japanese version of the Moral Sensitivity Questionnaire 2018; PHQ-9: Patient Health Questionnaire-9; GAD-7: Generalized Anxiety Disorder-7; SUB: Stress Underestimation Beliefs; NAQ-R: Negative Acts Questionnaire-Revised

## Discussion

To the best of our knowledge, this is the first study to examine the reliability and validity of the Japanese version of the TIC Provider Survey among health care workers. The results showed that the reliabilities were fair or excellent, except for knowledge, as with the original version. Regarding construct validity, the results were non-significant or significant but weak.

The reliability of the knowledge was not acceptable. Item-total correlation analysis of knowledge showed that items 2, 3, and 7 were not endorsed. (Appendix1) The items 2, 3, and 7 are reverse scoring items (Agree or Strongly Agree are wrong answers). Diagnostic criteria A of post-traumatic stress disorder (PTSD) and acute stress disorder in the Diagnostic and Statistical Manual of Mental Disorders, Fifth edition (DSM-5), involve exposure to life-threatening events [6]. Moreover, the diagnostic criteria and peritraumatic risk factors of PTSD or acute stress disorder in DSM-5 also involve, “the greater the magnitude of trauma, the greater the likelihood of PTSD,” and “the caused clinically significant distress or impairment in social, occupational, or other important areas of functioning” [6]. Therefore, it is not surprising for many healthcare workers to interrelate trauma experiences with developed trauma-related disorders, and it is thought that most people who experience severe trauma develop significant posttraumatic stress or PTSD. This might cause more than half of the participants to agree (choose wrong answer) with items 2, 3, and 7 (Appendix 2), which would lead to these items not endorsing the scale. However, the following are also described in the DSM-5: most people who experience trauma do not develop trauma-related disorders, and there are various factors, apart from the severity of trauma, to affect the traumatic stress reactions, as well as also some post traumatic symptoms, such as avoidance, which do not show obvious signs of distress. If participants had this knowledge, they could choose the correct answers for those items. The results demonstrate the need for greater learning about trauma among healthcare workers, apart from the education being provided in medical or nursing schools in Japan.

As for validity, moral sensitivity showed a moderate or weak correlation, as hypothesized. However, the correlations with other measures were not significant, or significant but weak, or the opposite of our hypotheses. There are several possible explanations for these results. ARTIC-10 assessed the attitude towards TIC, and might correlate with only the category considered similar to attitude towards TIC in the TIC Provider Survey, or the moderate internal consistency of ARTIC-10 might affect these weak correlations [27]. The lack of correlation between TIC Provider Survey, and PHQ-9 and GAD-7 might be due to differences in the sampling of studies. A previous study demonstrating a negative correlation between favorable attitudes towards TIC and burnout and STS, had recruited 1395 study participants from 17 settings with TIC programs [17]. Such settings have a TIC culture in their organizations. In this situation, favorable attitudes towards TIC are likely to be negatively associated with burnout and STS, because individuals could establish emotional safety by promoting self-care on a personal and organizational level through TIC training [28]. In contrast, the current study participants were not recruited on a hospital basis, so the TIC Provider Survey scores would not reflect the extent of TIC at their institutions. The negative correlation between self-rated competence and SUB might suggest consistency with the results of a previous study, showing that high competency was associated with confidence in one’s ability, which would lead to stress underestimation beliefs [24]. The positive correlation between self-rated competence and practice with NAQ-R might suggest consistency with the result of a previous study that showed that more capable workers are likely to be bullied because of jealousy [29, 30].

The present results suggested the necessity of specialized education on trauma and PTSD for the practical implementation of TIC in clinical settings. The TIC Provider Survey is anticipated to be valuable for visualizing the knowledge acquisition status of healthcare professionals necessary for TIC practice and enhancing educational content since it allows for assessing the effectiveness of corresponding education.

This study has some limitations. First, this study was conducted as an internet survey. Participants were selected from a database of people registered as monitors of the research company; those more concerned about TIC, quality of care for patients, and trauma were more likely to respond to the survey, thereby causing a sampling bias. Second, this study was conducted during the COVID-19 pandemic. In Japan, the second wave of coronavirus began in July 2020, and the number of people infected with the novel coronavirus marked a record high on November 18th, 2020 (2201 per day) [31]. A previous study showed that Japan’s healthcare workers experienced considerable psychological strain owing to the COVID-19 pandemic [32]. This could have resulted in an overestimation of psychological variables among the participants.

## Conclusions

We confirmed the reliability and examined the validity of the Japanese version of the TIC Provider Survey, among Japanese workers in a healthcare setting. The reliability of the scale was acceptable. Validity was modest or not acceptable.

## Electronic supplementary material

Below is the link to the electronic supplementary material.

Supplementary Material 1

## Data Availability

The data that support the findings of this study are available upon reasonable request.
