# Supplementary material for: Research note reliability and validity of Japanese version of the trauma-informed care provider survey (TIC provider survey)
Source: BMC Res Notes. 2023 May 2;16:68. doi: 10.1186/s13104-023-06337-8 (PMC10152421; doi:10.1186/s13104-023-06337-8)
Supplement: Supplementary file 1 — Supplementary Material 1 [file 13104_2023_6337_MOESM1_ESM.docx]

Appendix1. Item-total correlation analysis of Knowledge in the TIC provider survey (n = 794)

| Items of Knowledge | | r |
| --- | --- | --- |
| 1 | Almost everyone, who is seriously injured or ill, has at least one traumatic stress reaction in the immediate aftermath of the event. | 0.66 |
| 2 | It is inevitable that most individuals who experience a life-threatening illness or injury will go on to develop significant posttraumatic stress or PTSD. | -0.46 |
| 3 | Individuals who are more severely injured or ill generally have more serious traumatic stress reactions than those who are less severely injured or ill. | -0.50 |
| 4 | Individuals who, at some point during the traumatic event, believe that they might die are at greater risk for posttraumatic stress reactions. | 0.69 |
| 5 | Many individuals cope well on their own, after experiencing serious illness or injury. | 0.56 |
| 6 | The psychological effects of an injury or illness often last longer than the physical symptoms. | 0.73 |
| 7 | Individuals with significant posttraumatic stress reactions usually show obvious signs of distress. | -0.45 |
| 8 | I know the common signs and symptoms of traumatic stress in ill or injured patients. | 0.66 |
| 9 | Some early traumatic stress reactions in patients can be part of a healthy emotional recovery process. | 0.78 |
| 10 | There are things that providers can do to help prevent longer-term posttraumatic stress in ill and injured patients. | 0.78 |
| 11 | There are effective screening measures for assessing traumatic stress that providers can use in practice. | 0.68 |

Appendix 2. Percentage of endorsed items and correct answers to questions in the TIC provider survey (n = 794)

| Knowledge | | Strongly Disagree | Disagree | Agree | Strongly  Agree | Percentage  of correct answers (%) |
| --- | --- | --- | --- | --- | --- | --- |
| 1 | Almost everyone who is seriously injured or ill has at least one traumatic stress reaction in the immediate aftermath of the event. | 45 | 200 | 464 | 85 | 69.1 |
|  | (%) | 5.7 | 25.2 | 58.4 | 10.7 |  |
| 2 | It is inevitable that most individuals who experience a life-threatening illness or injury will go on to develop significant posttraumatic stress or PTSD. | 53 | 275 | 416 | 50 | 41.3 |
|  | (%) | 6.7 | 34.6 | 52.4 | 6.3 |  |
| 3 | Individuals who are more severely injured or ill generally have more serious traumatic stress reactions than those who are less severely injured or ill. | 46 | 223 | 473 | 52 | 33.9 |
|  | (%) | 5.8 | 28.1 | 59.5 | 6.6 |  |
| 4 | Individuals who, at some point during the traumatic event, believe that they might die are at greater risk for posttraumatic stress reactions. | 47 | 167 | 487 | 93 | 73.0 |
|  | (%) | 6.0 | 21.0 | 61.3 | 11.7 |  |
| 5 | Many individuals cope well on their own after experiencing serious illness or injury. | 75 | 353 | 338 | 28 | 46.1 |
|  | (%) | 9.4 | 44.5 | 42.6 | 3.5 |  |
| 6 | The psychological effects of an injury or illness often last longer than the physical symptoms. | 42 | 150 | 470 | 132 | 75.8 |
|  | (%) | 5.3 | 18.9 | 59.2 | 16.6 |  |
| 7 | Individuals with significant posttraumatic stress reactions usually show obvious signs of distress. | 45 | 270 | 421 | 58 | 39.7 |
|  | (%) | 5.7 | 34.0 | 53.0 | 7.3 |  |
| 8 | I know the common signs and symptoms of traumatic stress in ill or injured patients. | 63 | 301 | 393 | 37 | 54.2 |
|  | (%) | 7.9 | 37.9 | 49.5 | 4.7 |  |
| 9 | Some early traumatic stress reactions in patients can be part of a healthy emotional recovery process. | 41 | 172 | 526 | 55 | 73.2 |
|  | (%) | 5.1 | 21.7 | 66.3 | 6.9 |  |
| 10 | There are things that providers can do to help prevent longer-term posttraumatic stress in ill and injured patients. | 40 | 145 | 526 | 83 | 76.7 |
|  | (%) | 5.0 | 18.3 | 66.3 | 10.4 |  |
| 11 | There are effective screening measures for assessing traumatic stress that providers can use in practice. | 51 | 272 | 422 | 49 | 59.3 |
|  | (%) | 6.4 | 34.3 | 53.1 | 6.2 |  |

Note: Items 2, 3, and 7 are reverse-scoring items (Agree or Strongly Agree are wrong answers).
